# New York Desires a Higher Standard

**Published:** 1895-02

**Authors:** 


New York Desires a Higher Standard.—The proposed act, a summary of which is printed in this issue of The Journal, to complete veterinary legislation in New York State approaches the ideal very closely, and all movements in this direction looking toward a higher educational standard of the future veterinarian must surely command the strong support and potent influence of everyone who has at heart the true growth of the veterinary profession. Already the cry of alarm has been sounded against this act, because of the higher standard it demands and will establish. Let it be well considered and remembered in the counsels that will ultimately prevail that this has long and earnestly been pressing for solution, and those who may cry out against it to-day have for many years had in their hands the instruments to correct this evil. It may sound the death-knell of some institutions which for years have engrafted upon our country a class of graduates armed with diplomas that represented but little so far as a thorough education might be considered, and who for twenty or more years established so low a standard and were even willing to leave it forever thus, until strong arms of support were thrown around those few pioneers who had given the first impulses for higher veterinary education in America, and earnest, persistent agitation brought its just reward.

